# Choroidal Metastasis as the Initial Presentation of Lung Cancer: A Case Report

**DOI:** 10.7759/cureus.40955

**Published:** 2023-06-25

**Authors:** Alexandre Reis da Silva, Rita Basto, Renato Correia Barbosa, Ana Rita Viana, Carla Teixeira

**Affiliations:** 1 Ophthalmology Department, Hospital Pedro Hispano, Unidade Local de Saúde de Matosinhos, Matosinhos, PRT

**Keywords:** macular lesion, fluorescein angiography, intraocular metastasis, lung cancer, choroid

## Abstract

This case report presents an 82-year-old woman with decreased visual acuity. Clinical and multimodal evaluation revealed an elevated macular lesion with choroidal thickening. Fluorescein angiography and optical coherence tomography (OCT) showed characteristic patterns of choroidal metastasis. Thoracic CT scan confirmed a nodular mass in the right hilus, suggesting bronchogenic neoplastic process. A pleural biopsy demonstrated malignant neoplasia with characteristics of adenocarcinoma, and immunohistochemistry was positive for thyroid transcription factor 1 (TTF-1). These findings emphasize the critical role of ophthalmological examinations in early detection and intervention, as choroidal tissue is highly susceptible to metastasis, particularly from breast and lung cancers. Additionally, the poor prognosis associated with metastatic lung cancer underscores the urgency for prompt action.

## Introduction

The choroid is the most common place for intraocular metastasis [1]. Lung cancer accounts for the second most common cancer that leads to choroidal metastasis (21-29%), with breast cancer being the first [1,2]. Lung cancers that metastasize to the choroid are about 2-6.7% [3]; unilateral and unifocal masses are the most frequent finding associated with choroidal metastasis from lung cancer.

We present the case of an 82-year-old woman with choroidal metastasis as the initial presentation of lung cancer through the assessment of the clinical records and complementary exams (optical coherence tomography (OCT), ocular ultrasound, fluorescein angiography, and computed tomography (CT) scan).

## Case presentation

**Figure 1:**
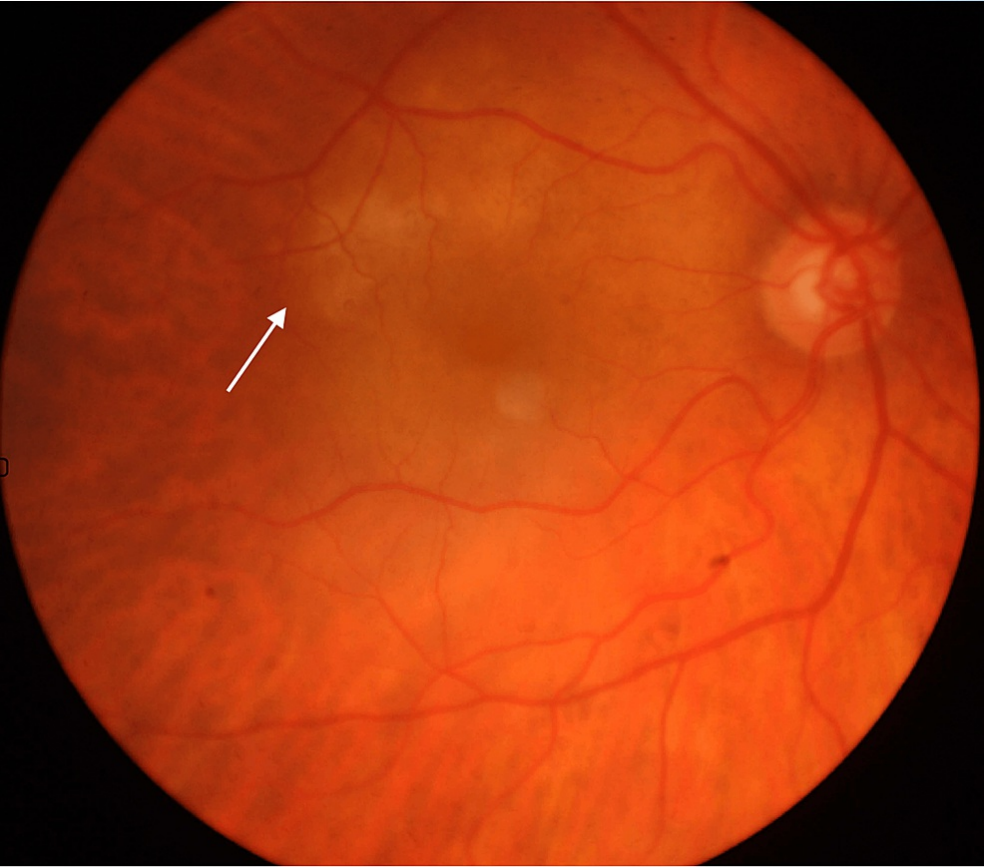
Yellow upper macular lesion demonstrated in retinography.

An 82-year-old woman who was subject to a Yttrium aluminum garnet (YAG) on the right eye presented to the emergency department due to maintenance of decreased visual acuity, with a best corrected visual acuity (BCVA) of 20/63. Fundus examination showed an elevated macular lesion (Figure 1). The left eye was normal.

**Figure 2:**
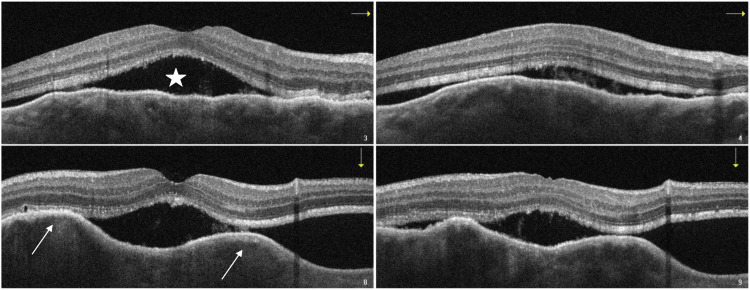
OCT section through the macula showing a choroidal elevation (arrows) with subretinal fluid (star). OCT: optical coherence tomography

**Figure 3:**
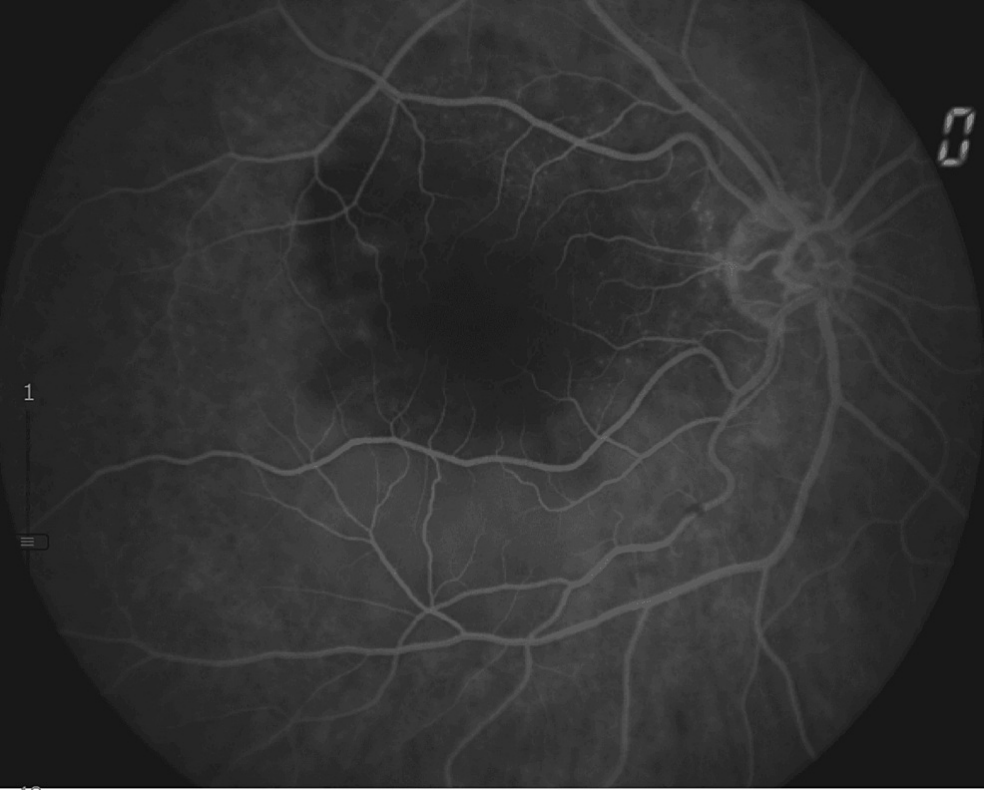
Choroidal hypofluorescence with hyperfluorescent pinpoint areas observed in the fluorescent angiography.

A complementary study of the right eye with OCT showed a choroidal elevation with a bumpy pattern and subretinal fluid (Figure 2). Fluorescent angiography demonstrated a yellow lesion superior to the macula with choroidal hypofluorescence and hyperfluorescent pinpoint areas (Figure 3).

**Figure 4:**
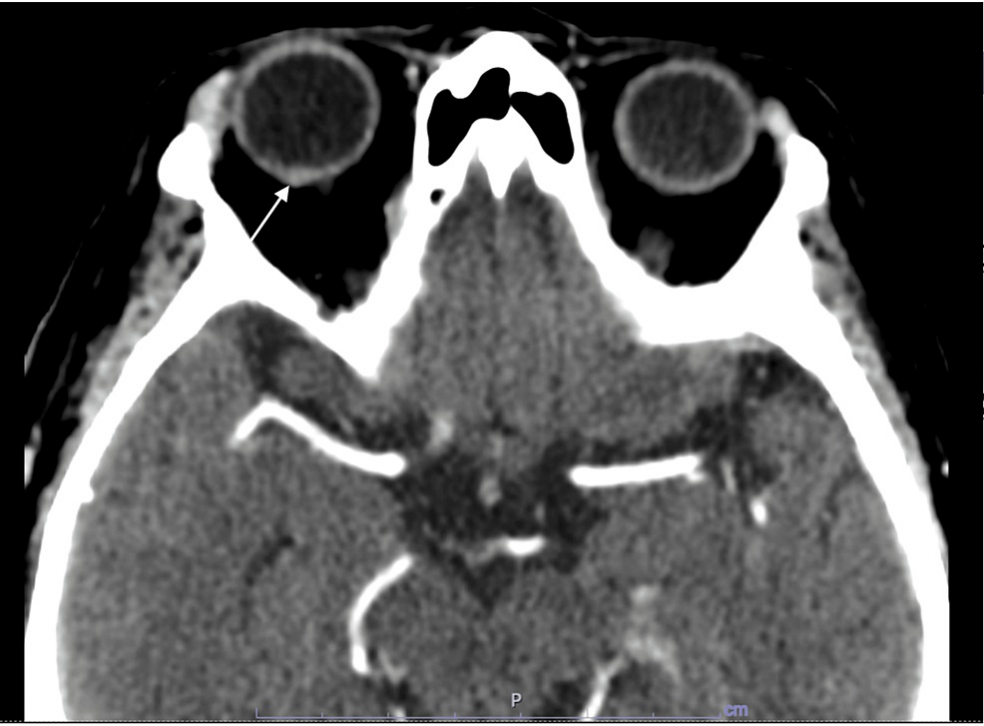
Orbit CT scan revealed a notable thickening of the choroid laterally to the optic nerve insertion. The use of contrast enhancement enhances the visibility of this distinct area of interest.

**Figure 5:**
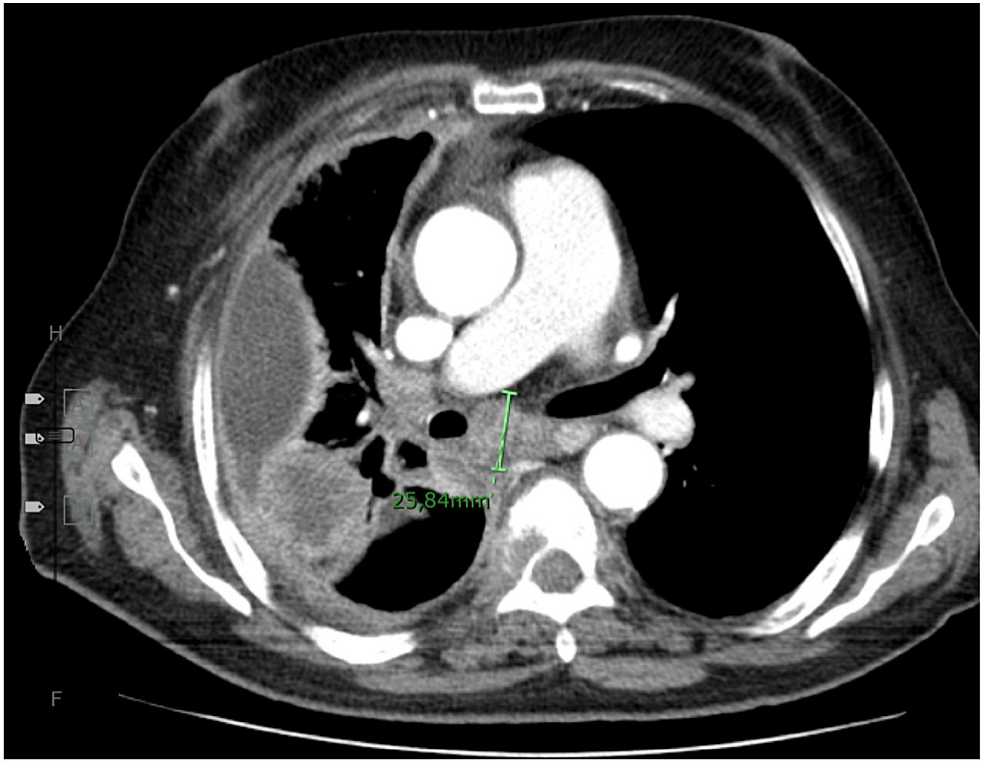
Thoracic section CT scan demonstrated a 2 cm nodular mass and pleural effusion that sugested a bronchogenic neoplastic process.

Additionally, she underwent an ocular ultrasound showing an elevated mass in the posterior pole of the eyeball that was consistent with a choroidal thickening with contrast enhancement shown in brain and orbit CT scan (Figure 4). Due to the high probability of choroidal metastasis, a thoraco-abdominopelvic CT scan was performed (Figure 5), which showed a voluminous pleural effusion and a 2 cm nodular mass in the right hilus, that suggested an undergoing bronchogenic neoplastic process.

Pleural biopsy confirmed an adenocarcinoma with positive thyroid transcription factor-1 (TTF-1) staining. Next-generation sequencing identified a mutation in the *HER2* gene (c.2313_2324dup) but no mutations in *EGFR*, *KRAS*, or *BRAF* genes were found.

With a diagnosis of stage IV non-small cell lung cancer with choroidal and pleural metastasis, the patient was referred for palliative care in the Oncology and Internal Medicine departments. No specific ophthalmologic treatment was administered for the choroidal mass. Unfortunately, the patient passed away five months after the initial diagnosis.

## Discussion

Intraocular metastasis most commonly affects the uveal tissue [4], with the choroidal tissue being the most frequently involved site, accounting for approximately 88% of uveal metastases [2]. The uveal tract's rich vascularity provides an ideal environment for cancer cells to access and colonize this tissue [5]. Breast cancer and lung cancer emerge as the two primary cancer types associated with choroidal metastasis, with breast cancer comprising 40-47% of cases and lung cancer accounting for 21-29% [1].

Our findings are consistent with previous reports indicating that choroidal metastasis from lung cancer often presents as a unifocal and unilateral pattern of metastasis [2]. This pattern aligns with the observations made in our case report, highlighting the concordance with existing literature. Fluorescein angiography serves as a valuable diagnostic tool for choroidal metastases. During the early arterial phases of fluorescein angiography, choroidal metastases typically display a hypofluorescent pattern, in contrast to the subsequent hyperfluorescence observed during the venous phases [1]. Additionally, the presence of dilated retinal capillaries with pinpoint leakage at the tumor border is a characteristic finding in approximately 73% of cases of choroidal metastases [6].

The prognosis for patients with choroidal metastasis from lung cancer is generally poor, as the presence of such metastases signifies a hematogenous spread of the cancer [7]. Studies have reported a one-year mortality rate of 54% from the time of detection of lung cancer uveal metastasis, indicating the aggressive nature of the disease [8].

## Conclusions

Our findings corroborate the well-established understanding that uveal tissue, particularly the choroid, is highly susceptible to intraocular metastasis. Decreased visual acuity in a patient with a macular lesion and a choroidal hypofluorescent pattern is frequently associated with choroidal metastasis from a primary occult cancer. Breast cancer and lung cancer represent the most common primary malignancies leading to choroidal metastasis. Given the poor prognosis associated with lung cancer uveal metastasis, early detection and prompt intervention are crucial for optimizing patient outcomes.
